# Characterization of a nuclear transport factor 2-like domain-containing protein in *Plasmodium berghei*

**DOI:** 10.1186/s12936-024-04839-9

**Published:** 2024-01-09

**Authors:** Mamoru Niikura, Toshiyuki Fukutomi, Jiro Mitobe, Fumie Kobayashi

**Affiliations:** 1 Department of Infectious Diseases, Kyorin University School of Medicine, Tokyo, Japan; 2 Department of Pharmacology and Toxicology, Kyorin University School of Medicine, Tokyo, Japan; 3 Department of Environmental Science, School of Life and Environmental Science, Azabu University, Kanagawa, 252-5201 Japan

**Keywords:** *Plasmodium*, NTF2-like protein, Noncanonical, mRNA, Export

## Abstract

**Background:**

*Plasmodium* lacks an mRNA export receptor ortholog, such as yeast Mex67. Yeast Mex67 contains a nuclear transport factor 2 (NTF2)-like domain, suggesting that NTF2-like domain-containing proteins might be associated with mRNA export in *Plasmodium.* In this study, the relationship between mRNA export and an NTF2-like domain-containing protein, PBANKA_1019700, was investigated using the ANKA strain of rodent malaria parasite *Plasmodium berghei*.

**Methods:**

The deletion mutant Δ*1019700* was generated by introducing gene-targeting vectors into the *P. berghei* ANKA genome, and parasite growth and virulence were examined. To investigate whether PBANKA_1019700 is involved in mRNA export, live-cell fluorescence imaging and immunoprecipitation coupled to mass spectrometry (IP-MS) were performed using transgenic parasites expressing fusion proteins (1019700::mCherry).

**Results:**

Deletion of PBANKA_1019700 affected the sexual phase but not the asexual phase of malaria parasites. Live-cell fluorescence imaging showed that PBANKA_1019700 localizes to the cytoplasm. Moreover, IP-MS analysis of 1019700::mCherry indicated that PBANKA_1019700 interacts with ubiquitin-related proteins but not nuclear proteins.

**Conclusions:**

PBANKA_1019700 is a noncanonical NTF2-like superfamily protein.

**Supplementary Information:**

The online version contains supplementary material available at 10.1186/s12936-024-04839-9.

## Background

Export of mRNA through nuclear pores into the cytoplasm is an essential process in all eukaryotic cells. The steps of mRNA export have mostly been studied in opisthokonts, such as yeast [1, 2]. Mature mRNA is recognized by adaptor proteins such as nuclear poly(A) binding protein 2 (NAB2), yeast RNA annealing protein (YRA1), and three serine/arginine-rich (SR) proteins, including nucleolar protein 3 (NPL3), G-strand binding protein 2 (GBP2) and hypothetical RNA-binding protein (HRB1) [1, 3]. The mRNA export receptor, Mex67/Mtr2, recruited by these adaptor proteins interacts with nuclear pore complex proteins to export mRNA into the cytoplasm [2, 4, 5].

*Plasmodium* is a unicellular eukaryote with non-photosynthetic plastids that belongs to the phylum Apicomplexa [6]. *Plasmodium* possess orthologs of yeast adaptor proteins, but no orthologs of Mex67 and Mtr2 [7–9]. The mRNA export receptor in plants remains undiscovered [10]. The mechanism of mRNA export in eukaryotes other than opisthokonts, such as yeasts and humans, is currently unclear.

In a previous study, mRNA export receptor-associated protein was not detected by immunoprecipitation coupled to mass spectrometry (IP-MS) in *Plasmodium berghei* [7], but its existence was not definitively ruled out. An *in silico* analysis may yet identify it in malaria parasites. Yeast Mex67 contains a nuclear transport factor 2 (NTF2)-like domain [11]. The NTF2-like domain is necessary for heterodimerization with Mtr2 and for interaction with FG nucleoporins in the nuclear pores [11, 12]. Based on these findings, an NTF2-like domain-containing protein might be associated with mRNA export in *Plasmodium.*

In the PlasmoDB database (www.plasmodb.org/), three proteins containing NTF2-like domains, namely, PBANKA_1019700 (conserved *Plasmodium* protein), PBANKA_0922900 (mitochondrial import inner membrane translocase subunit TIM44), and PBANKA_1030300 (nuclear transport factor 2), have been annotated. PBANKA_1030300 and PBANKA_0922900 are essential to parasite survival during the erythrocytic stage. PBANKA_1030300 binds RanGDP and is associated with protein import to the nucleus from the cytoplasm [13]. PBANKA_0922900 contains a Tim44-like domain, indicating that it localizes to the mitochondria, not to the nucleus of *Plasmodium*. PBANKA_1019700 remains poorly understood, but in *P. berghei* it might be the mRNA export receptor-associated protein. In the present study, this possibility was investigated by examining the role and localization of PBANKA_1019700 by gene deletion, live-cell fluorescence imaging, and IP-MS.

## Methods

### Mouse studies and ethics

Five- to six-week-old female C57BL/6J (B6) mice were purchased from CLEA Japan Inc. (Tokyo, Japan). The experiments were approved (#221) by the Experimental Animal Ethics Committee of Kyorin University School of Medicine (Tokyo, Japan), and all experimental animals were kept at the animal facility in a specific-pathogen-free unit with sterile bedding, food, and water.

The infection studies included frequent observations to determine humane endpoints, at which mice were unable to ambulate sufficiently to obtain water or food. At the indicated time points, mice were euthanized by cervical dislocation under isoflurane anesthesia. All experiments were designed to minimize suffering. When symptoms or death was expected due to experimental infections, mice were visually checked by investigators at least twice daily (including weekends and holidays). Mice that exhibited signs of neurological distress, such as cerebral paralysis, were humanely sacrificed by cervical dislocation under isoflurane anesthesia and scored as deaths. No mice died before meeting the criteria for euthanasia. The investigators who conducted the experiments had completed the Experimental Animal Ethics Committee training course on animal care and handling.

### Parasites and infection

Malaria parasites were stored as frozen stocks in liquid nitrogen. Luciferase-expressing *P. berghei* (strain ANKA) were generated as previously described [14, 15]. Erythrocytes parasitized with transfected parasites were generated in donor mice inoculated intraperitoneally with frozen stocks of parasites. The donor mice were monitored for parasitaemia daily and bled for experimental infection during periods in which the level of parasitaemia increased (1–2% parasitaemia). Experimental mice were infected intravenously with 1 × 10^4^ parasitized erythrocytes or 5 × 10^6^ to 5 × 10^7^ purified mature schizonts harvested by Nycodenz density gradient centrifugation of infected blood from a given parasite strain.

### Transfection

To generated deletion mutants of *P. berghei* ANKA, the gene-targeting vectors for *PBANKA_1019700*, *PBANKA_1101300* (*sbp1*) and *PBANKA_0519900* were designed and constructed as shown in Additional file 1: Fig. S1. Briefly, the 5′ and 3′ flanking regions of the open reading frame (ORF) of target genes were amplified by PCR. The PCR products were annealed to either side of the *human dihydrofolate reductase* (*hdhfr*)-expressing cassette, the red fluorescent protein gene (*mCherry*)-*hdhfr*-expressing cassette or the luciferase gene (*luc2*)-*hdhfr*-expressing cassette and amplified by PCR using gene-specific primers (Additional file 1: Fig. S1 and Additional file 2: Table S1) as previous studies [16, 17]. The gene-targeting vectors were introduced into the 5′ and 3′ flanking regions of target genes by double-crossover homologous recombination. To generate transgenic parasites expressing mCherry-fused PBANKA_1019700, the gene-targeting vectors for PBANKA_1019700 were prepared by PCR (Additional file 1: Fig. S1). The PCR products were annealed to either side of the red fluorescent protein gene (*mCherry*)-*hdhfr*-expressing cassette and amplified by PCR using gene-specific primers (Additional file 1: Fig. S1 and Additional file 2: Table S1). The gene-targeting vectors were introduced into the 3′ flanking regions of target genes by double-crossover homologous recombination.

To generate transgenic parasites expressing GFP-fused PBANKA_0519900 and GFP-fused PBANKA_1359300, the gene-targeting vectors for PBANKA_0519900 and PBANKA_1359300 were prepared by PCR, respectively (Additional file 1: Fig. S1). The PCR products were annealed to either side of the green fluorescent protein gene (*gfp*)-mutated *human deoxyhypusine synthase* (*hdhps*)-expressing cassette [18] and amplified by PCR using gene-specific primers (Additional file 1: Fig. S1 and Additional file 2: Table S1). The gene-targeting vectors were introduced into the 3′ flanking regions of ORFs of target genes by double-crossover homologous recombination. Transfection was performed using an Amaxa Basic Parasite Nucleofector Kit (Amaxa GmbH, Cologne, Germany) according to the manufacturer’s protocol [19].

### Genomic PCR

To generate gene-targeting vectors and confirm the introduction of gene-targeting vectors into target genes, genomic PCR was performed as described previously [7]. Thirty-five cycles of PCR were performed on a C1000 thermal cycler (Bio-Rad, Hercules, CA, USA). Each cycle consisted of denaturation at 98 °C for 15 s, annealing at 55 °C for 15 s, and extension at 68 °C for 1–6 min. The PCR products were then analysed on a 1% (w/v) agarose gel and stained with ethidium bromide.

### Parasitaemia

Methanol-fixed tail-blood smears, stained with 3% Giemsa diluted with phosphate buffer (pH 7.2) for 45 min, were subjected to microscopic examination. The number of parasitized erythrocytes (out of 250 erythrocytes) was enumerated when the level of parasitaemia and gametocytaemia exceeded 10%, while 1 × 10^4^ erythrocytes were examined in mice with lower levels of parasitaemia and gametocytaemia. The parasitaemia and gametocytaemia percentage values were calculated as follows: [(number of parasitized erythrocytes) ÷ (total number of erythrocytes)] × 100.

### Evaluation of gametocyte production in vitro

To evaluate gametocyte production, early trophozoite stage malaria parasites were obtained from B6 mice exhibiting 1–2% parasitaemia. Parasitized erythrocytes were incubated for 22 h, 28 h, 45 h in a 12-well plate. Methanol-fixed blood smears, stained with 3% Giemsa diluted in phosphate buffer (pH 7.2) for 45 min, were subjected to microscopic examination. Erythrocytes parasitized with mature schizonts containing 4–15 merozoites, and mature gametocytes showing sex-specific features such as nuclear enlargement, were counted as described previously [7, 17]. The distribution of pigment granules throughout the cytoplasm, and enlargement of cells, were also assessed [7, 17]. The proportions of male and female gametocytes were determined in at least 300 parasitized erythrocytes. The proportions of male and female gametocytes were calculated as follows: [(number of male or female gametocytes) ÷ (total number of schizonts plus male and female gametocytes)] × 100.

### Ex vivo organ bioluminescence imaging

Bioluminescence imaging was performed using a Photon IMAGER system (Biospace Lab, Nesles-la-Vallée, France) as previously described [15]. Mice were administered 1.5 mg of VivoGlo™ Luciferin (In Vivo Grade, Promega, Japan) dissolved in 150 µL of phosphate buffered saline by intravenous injection. At 15 min after receiving the VivoGlo™ Luciferin, organs were collected for image acquisition within 5 min. A charge-coupled device camera was used to monitor the acquisition of emitted photons. Ex vivo bioluminescence imaging data were analysed using the M3 software (Biospace) with size-constant regions of interest (ROIs).

### Fluorescence live cell imaging

Nuclear DNA was stained using Hoechst 33342 dye (Invitrogen, Waltham, MA). To examine the localization of PBANKA_1019700::mCherry, Hoechst 33342 was added to the culture of parasitized erythrocytes at a concentration of 1 µg/mL. The staining medium was removed after the incubation, and fresh RPMI1640 medium was added. Brightfield and fluorescence micrographs were captured at 1000× magnification using an All-in-One Fluorescence Microscope (BZ-X800; KEYENCE Japan, Osaka, Japan).

### Protein IP

Parasitized erythrocytes were transferred to RPMI1640 medium supplemented with 25% fetal bovine serum, 0.05 mg/mL penicillin and 0.05 mg/mL streptomycin. The parasitized erythrocytes were incubated for 22 h in 90% N_2_, 5% CO_2_ and 5% O_2_. Mature schizonts and gametocytes were harvested by Nycodenz density gradient centrifugation, as described previously [7]. Proteins were extracted using Mammalian Protein Extraction Reagent (Thermo Fisher Scientific, Waltham, MA) according to the manufacturer’s protocol. Protein IP in transgenic parasites expressing mCherry fused to PBANKA_1019700 was performed using RFP-Trap Agarose and a RFP-Trap-A kit, according to the manufacturer’s instructions (Chromotek, Planegg, Germany).

### Mass spectrometry

All of the fractionated peptides obtained according to the manufacturer’s instructions of RFP-Trap-A kit were injected into a trap column (C18, 75 μm × 2 cm; Acclaim PepMap 100, Thermo Fisher Scientific) and an analytical column (C18, 0.075 × 120 mm; Nikkyo Technos, Tokyo, Japan), which was attached to a nano liquid chromatography-tandem mass spectrometry (nanoLC-MS/MS) system. The nanoLC-MS/MS analysis was conducted using an Q Exactive plus mass spectrometer (Thermo Fisher Scientific) equipped with a nanoLC interface and a nano high-performance liquid chromatography (nanoHPLC) system (EASY-nLC 1200, Thermo Fisher Scientific). Purified peptides from the nanoLC were introduced into the Q Exactive plus, a hybrid quadrupole Fourier transform mass spectrometer. Full MS and MS/MS scans were followed by higher energy collisionally activated dissociation (HCD). The database search engines Proteome Discoverer 1.4 (Thermo Scientific) and MASCOT 2.6 (Matrix Science) were used to identify and quantify proteins from the MS, MS/MS and reporter ion spectra of the peptides. Peptide mass data were matched by searching the protein database (PlasmoDB-59_PbergheiANKA.fasta), downloaded from PlasmoDB (updated August 22, 2022). The false discovery rate (FDR) [20] was calculated by peptide sequence analysis using Percolator software [21]. High-confidence peptide identifications were obtained by setting a target false discovery rate threshold of ≤ 1.0% at the peptide level. Proteins exhibiting at least three peptide spectral matches were included.

### Statistical analysis

For time-series comparisons, Student’s t test was performed using Statcel program (OMS, Saitama, Japan). Survival curves were compared using a log-rank test. P-values < 0.05 were considered statistically significant.

## Results

### The NTF2 domain-containing protein PBANKA_1019700 is not associated with asexual development in malaria parasites

**Fig. 1:**
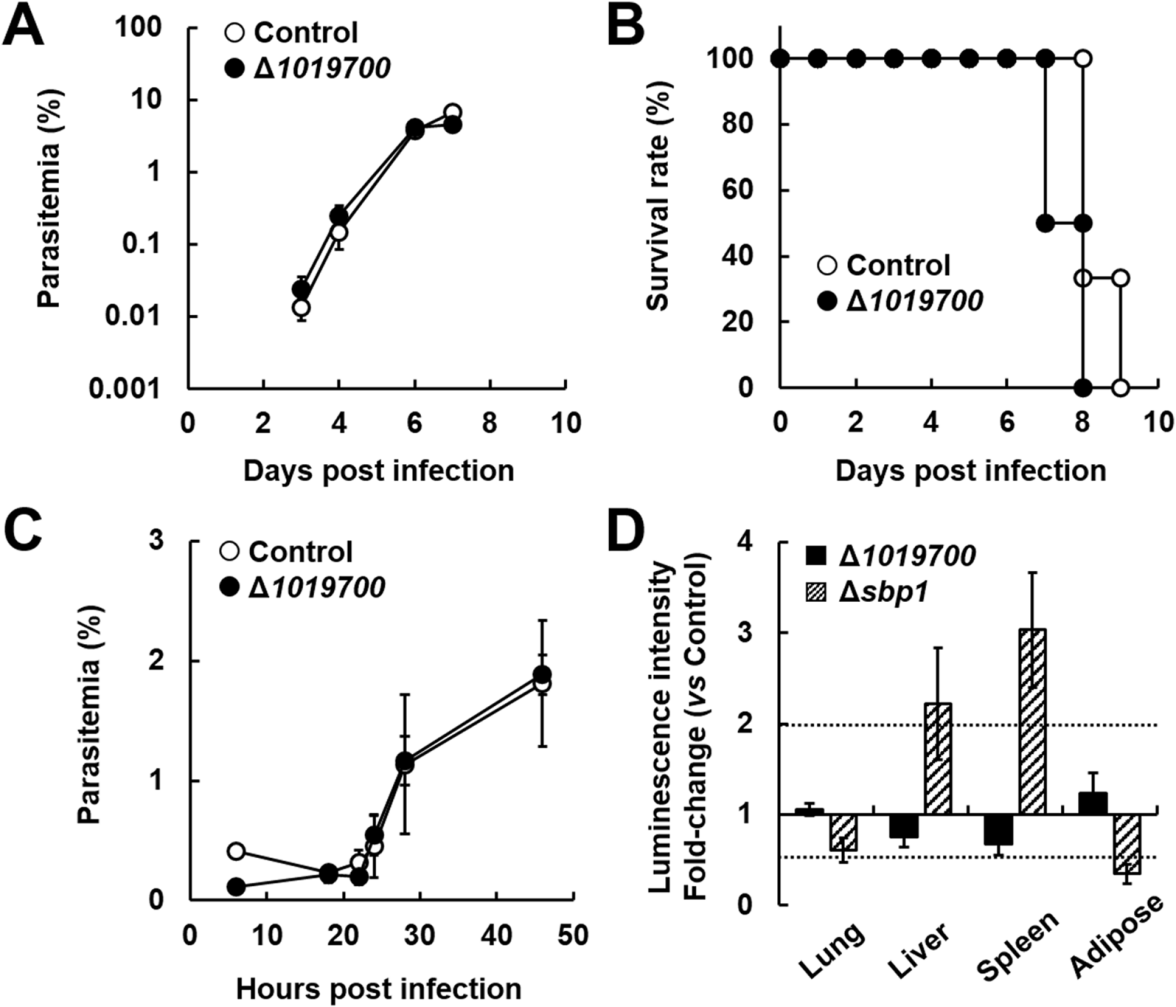
Effect of PBANKA_1019700 deletion on the asexual and sexual development of *Plasmodium berghei* (strain ANKA). Female C57BL/6 (B6) mice were infected with 1 × 10^4^ erythrocytes parasitized with *Plasmodium berghei* ANKA with *PBANKA_1019700* deletion (Δ*1019700*). As a control, *P. berghei* ANKA with *p230* deletion was inoculated intravenously into mice. **A** Time course of parasitaemia. Results are expressed as mean ± standard deviation (SD) from six mice. Experiments using six mice were performed in triplicate. **B** Survival rate. Data are the mean ± SD from six mice and are representative of three independent experiments. **C** Cell cycle during the asexual phase. Results are mean ± SD from three independent experiments. **D** Bioluminescence images of luciferase activity in the organs of infected mice. For bioluminescence analysis, erythrocytes parasitized with *Pb* ANKA were transferred to RPMI1640 medium supplemented with 25% fetal bovine serum, 0.05 mg/mL penicillin, and 0.05 mg/mL streptomycin. The parasitized erythrocytes were incubated for 18 h under 90% N_2_, 5% CO_2_, and 5% O_2_. Mature schizonts and gametocytes were harvested using Nycodenz density gradient centrifugation. B6 mice were injected with 5 × 10^6^–5 × 10^7^ schizonts of luciferase-expressing *P. berghei* with *p230* deletion (control), Δ*1019700* or Δ*sbp1* mutants. At 22 h post-infection, d-luciferin (1.5 mg) was injected into the tail vein of each mouse and the organs from each group were removed after perfusion. Fold change indicates the change in luciferase activity compared to mice infected with luciferase-expressing *P. berghei* with *p230* deletion (control). Dotted lines indicate significant differences (≥ twofold or ≤ 0.5-fold). Results are expressed as mean ± SD from three mice. Experiments were performed in duplicate with similar results

To investigate the relationship between mRNA export and PBANKA_1019700, deletion mutants (Δ*1019700*) were generated by introducing gene-targeting vectors into the *P. berghei* ANKA genome (Additional file 1: Fig. S1A). The course of parasitaemia in mice infected with Δ*1019700* mutants was comparable to that in mice infected with control parasites (Fig. 1A). On day 8 post-infection, all mice infected with Δ*1019700* mutants died, which was comparable to mice infected with control parasites (Fig. 1B). To examine the effects of PBANKA_1019700 deletion on the cell cycle during the asexual phase, mice were injected with schizonts of the control parasites and Δ*1019700* mutants. At 22 h after schizont injection, ring stage-infected erythrocytes were observed in mice treated with both the control and Δ*1019700* parasites (Fig. 1C).

Schizonts of malaria parasites bind to vascular endothelial cells through interactions between the endothelial receptor CD36 and sequestration-related proteins such as skeleton binding protein 1 (SBP1) and membrane associated histidine-rich protein 1a (MAHRP1a) [22]. To investigate whether the accumulation pattern of parasitized erythrocytes in the host is altered by PBANKA_1019700 deletion, bioluminescence imaging was performed (Fig. 1D, Additional file 1: Fig. S1B). The accumulation pattern of parasitized erythrocytes was not altered by PBANKA_1019700 deletion (Fig. 1D). Meanwhile, mutant *P. berghei* ANKA lacking SBP1 (Δ*sbp1*), which was generated as a positive control, exhibited reduced sequestration in the lung and adipose tissues of mice (Fig. 1D, Additional file 1: Fig. S1C). Together, these results show that PBANKA_1019700 deletion does not affect the asexual development of malaria parasites.

Male and female gametocyte production is reduced in Δ*1019700* mutants

### Male and female gametocyte production is reduced in Δ*1019700* mutants

**Fig. 2:**
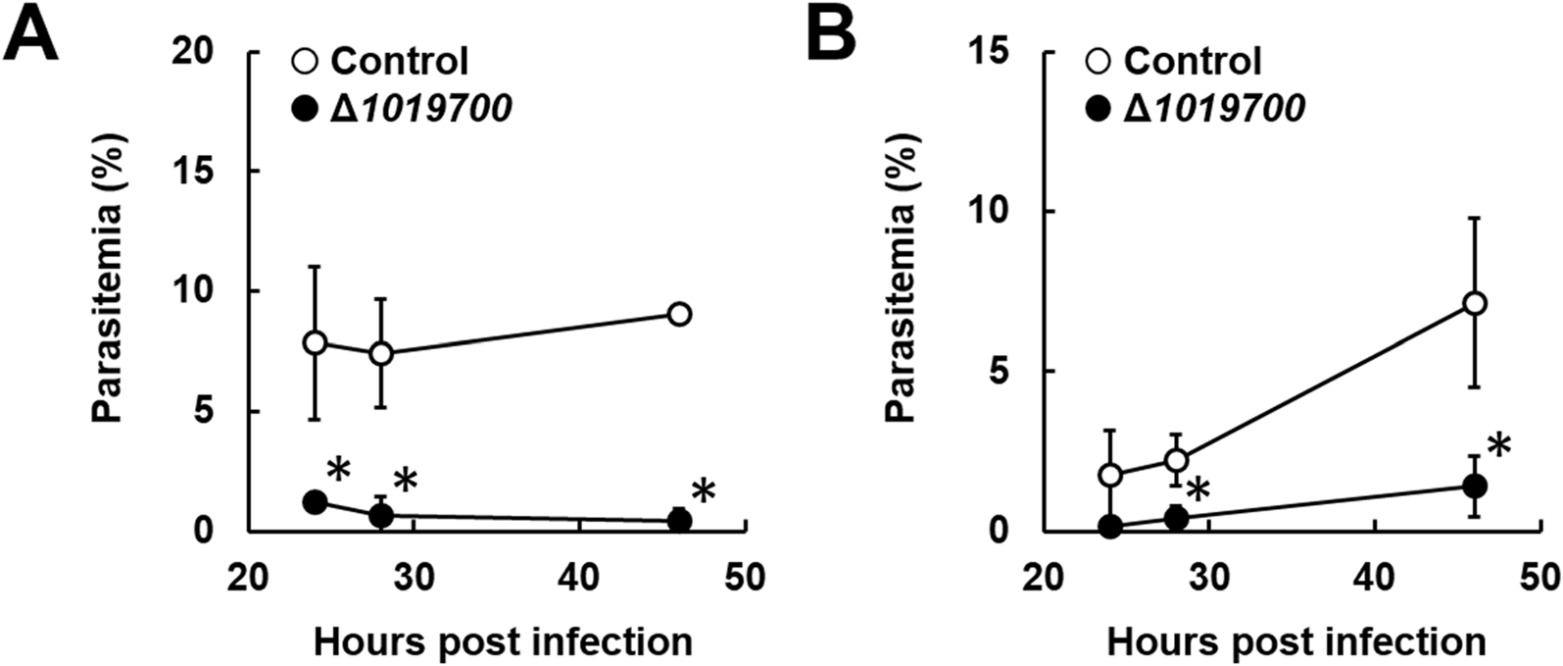
Effect of *PBANKA_1019700* deletion on the sexual development of *P. berghei* ANKA. Erythrocytes parasitized with *Plasmodium* were incubated for 22 h, 28 h, and 45 h. The percentages of male and female gametocytes were calculated as follows: ([number of male or female gametocytes] ÷ [total number of schizonts plus male and female gametocytes]) × 100. **A** Percentages of mature female gametocytes. **B** Percentages of mature male gametocytes. Results are expressed as the mean ± SD from three independent experiments. *Significant difference compared to controls

To investigate the effects of PBANKA_1019700 deletion on gametocyte production in malaria parasites, erythrocytes parasitized with the control parasites and Δ*1019700* mutants were cultured for 22, 28, or 45 h. The percentages of male and female gametocytes were lower in cultured Δ*1019700* mutants than in control parasites (Fig. 2). These findings indicate that the NTF2 domain-containing protein PBANKA_1019700 is involved in male and female gametocyte production in malaria parasites.

### Cellular localization of PBANKA_1019700 in malaria parasites

**Fig. 3:**
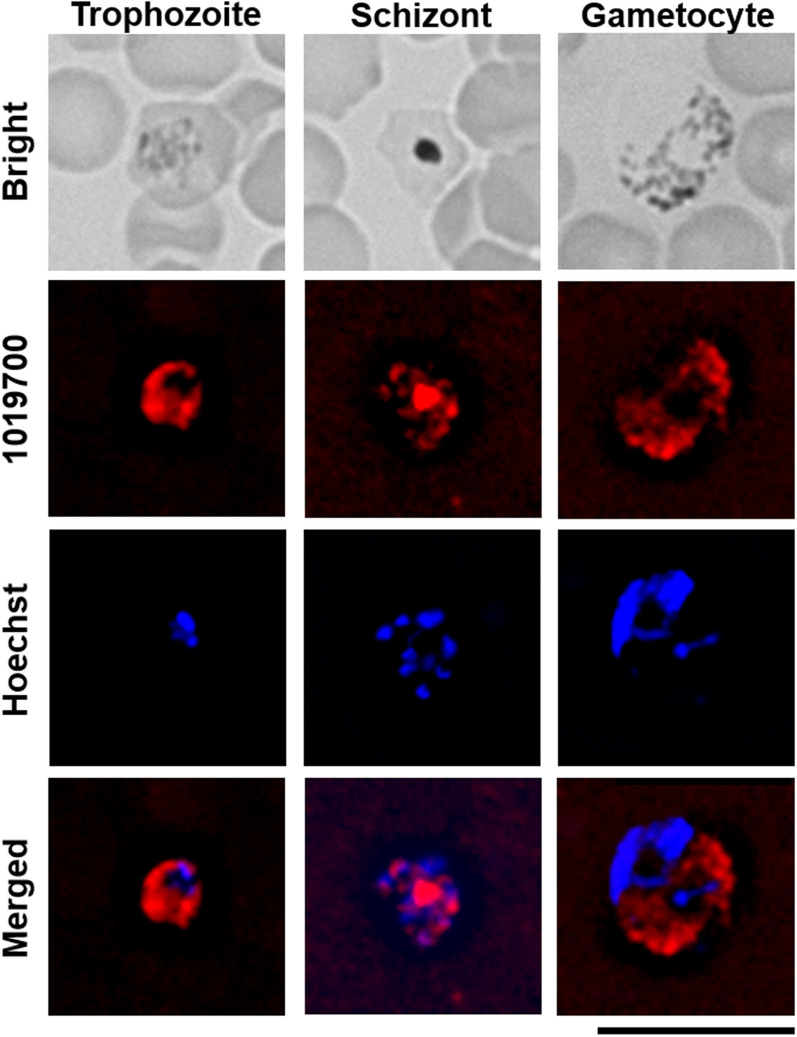
Live-cell fluorescence imaging of 1019700::mCherry-expressing parasites. Female B6 mice were infected with 1 × 10^4^ erythrocytes parasitized with transgenic *Plasmodium* expressing mCherry fused to PBANKA_1019700 (red). The cells’ nuclei were stained with Hoechst 33342 (blue). At least 50 parasitized erythrocytes were analysed, and the same fluorescence pattern was observed in all parasitized erythrocytes. Representative data are shown. Scale bar = 10 μm

To investigate whether the NTF2 domain-containing protein PBANKA_1019700 is involved in mRNA export, transgenic parasites expressing the fusion protein 1019700::mCherry were generated (Additional file 1: Fig. S2) and the cellular localization of 1019700::mCherry was analysed. The mCherry tag was introduced at the C-terminus of endogenous *PBANKA_1019700*. *1019700::mCherry* expression was controlled by the endogenous native promoters. Transgenic parasites expressing 1019700::mCherry (1019700::mCherry mutants) were successfully generated (Additional file 1: Fig. S2B, C) and found to express the fusion protein (Table 1; Fig. 3). Live-cell fluorescence imaging detected mCherry fluorescence in all erythrocytic stages, including trophozoites, schizonts, and gametocytes, of 1019700::mCherry mutants (Fig. 3). The observed mCherry fluorescence was localized to the cytoplasm of 1019700::mCherry mutants (Fig. 3). These results suggest that PBANKA_1019700 is a cytoplasmic protein.

**Table 1:** Results of immunoprecipitation coupled with mass spectrometry for 1019700::mCherry parasites

Accession #	Description	Σ# PSMs	# AAs	MW [kDa]	Calc. pI
PBANKA_1019700	Conserved *Plasmodium* protein, unknown function	1098	1176	136.8	8.50
PBANKA_0519900	Conserved *Plasmodium* protein, unknown function	212	1591	180.2	9.25
PBANKA_1359300	VPS13 domain-containing protein, putative	209	5434	629.9	7.64
PBANKA_0415500	Coatomer subunit beta, putative	188	999	116.5	5.90
PBANKA_0210600	Ubiquitin carboxyl-terminal hydrolase, putative	181	2788	326.0	8.48
PBANKA_1012800	Voltage-dependent anion-selective channel protein, putative	175	289	33.3	9.17
PBANKA_0207000	Calcium-transporting ATPase, putative	172	1120	127.3	7.53
PBANKA_0823000	Ubiquitin-like protein, putative	165	1384	161.4	8.66
PBANKA_1438300	Phospholipid-transporting ATPase, putative	164	1603	187.7	8.76
PBANKA_0316200	*Plasmodium* exported protein, unknown function	161	1739	206.1	3.97
PBANKA_1310800	40S ribosomal protein S5, putative	158	271	29.9	10.05
PBANKA_0802300	HECT-type E3 ubiquitin ligase UT, putative	150	3439	405.1	7.91

Proteins were extracted from 1019700::mCherry schizont- and gametocyte-enriched cultures after 22 h of culturing. Proteins with 50 peptide spectral matches (averaged across three independent experiments) and a fold change ≥ 5 compared to controls are listed. Control experiments involved immunoprecipitation of wild-type *P. berghei* ANKA using anti-mCherry beads coupled to mass spectrometry. Experiments were performed in triplicate. The results presented are sums from three independent experiments

*PSMs* peptide spectral matches, *AAs* amino acids, *MW* molecular weight, *Calc. pI* calculated isoelectric point

### Identification of PBANKA_1019700-interacting proteins in malaria parasites

To investigate the proteins that interact with the NTF2 domain-containing protein PBANKA_1019700, protein immunoprecipitation (IP) using anti-mCherry beads was performed and the proteins bound to PBANKA_1019700 were identified through mass spectrometry (MS). IP-MS using anti-mCherry beads with wild-type *P. berghei* ANKA was performed as a control.

In three independent comparative proteomics analyses, 1244, 1304, and 1183 proteins were detected. Among them, 12 proteins with at least 50 peptide spectral matches (averaged from three independent experiments) and a fold change ≥ 5 compared to the control in three independent experiments were analysed further (Table 1 and Additional file 3: Table S2). Cytoplasmic proteins, including ubiquitin-related proteins (ubiquitin carboxyl-terminal hydrolase, PBANKA_0210600; ubiquitin-like protein, PBANKA_0823000; HECT-type E3 ubiquitin ligase UT, PBANKA_0802300) and a VPS13 domain-containing protein (PBANKA_1359300) were detected through IP-MS of 1019700::mCherry (Table 1). However, RNA-binding proteins, such as NAB2 and GBP2, were not detected.

### The conserved *Plasmodium* protein PBANKA_0519900 is associated with asexual development in malaria parasites

**Fig. 4:**
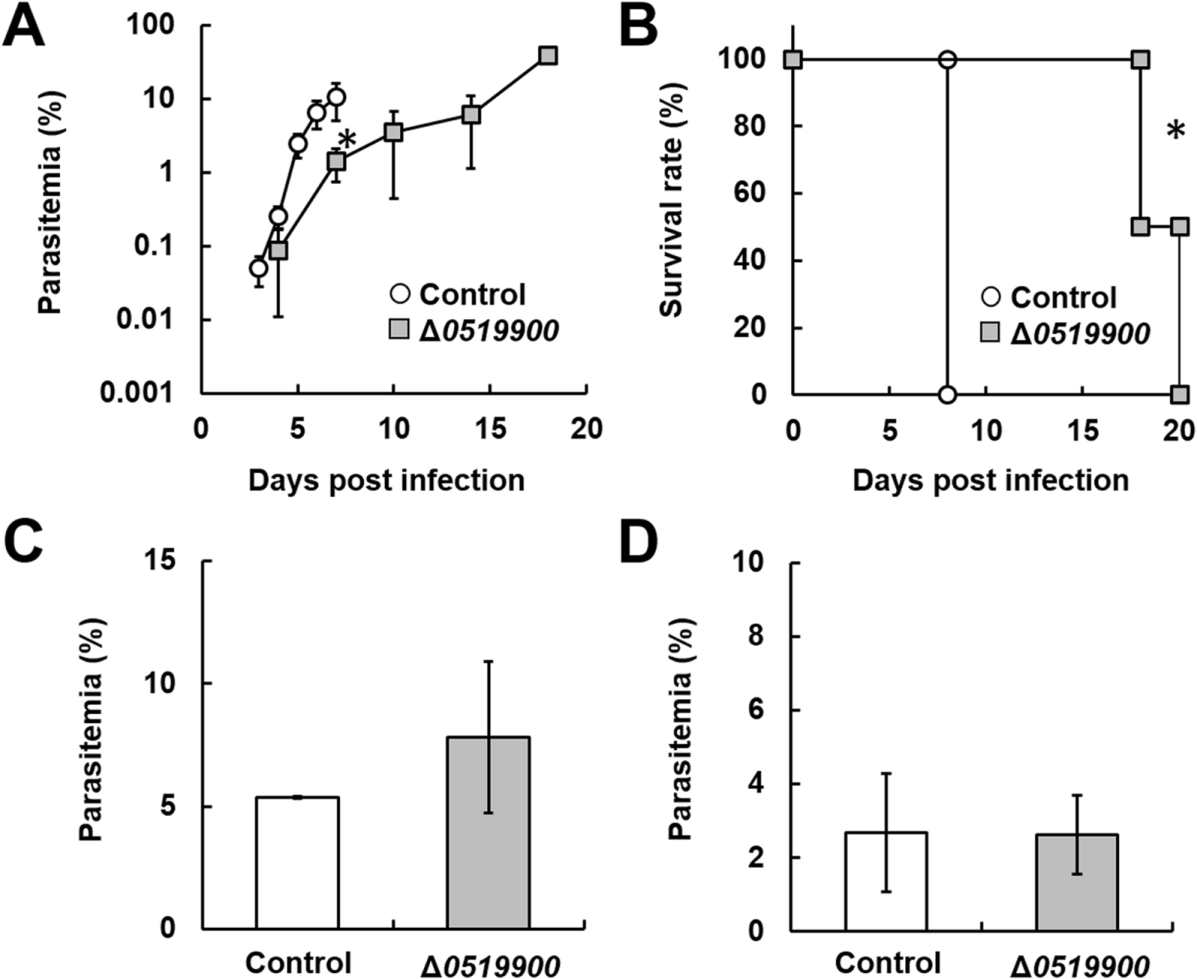
Effect of PBANKA_0519900 deletion on the asexual and sexual development of *P. berghei* ANKA. Female B6 mice were infected with 1 × 10^4^ erythrocytes parasitized with *P. berghei* ANKA with *PBANKA_0519900* deletion (Δ*0519900*). As a control, *P. berghei* ANKA with *p230* deletion was inoculated intravenously into mice. *Significant difference compared to controls. **A** Time course of parasitaemia. Results are expressed as mean ± SD from four mice. Experiments using four mice were performed in triplicate. **B** Survival rate. Data are the mean ± SD from four mice and are representative of three independent experiments. **C**, **D** Percentages of mature female (**C**) and male (**D**) gametocytes. Erythrocytes parasitized with *Plasmodium* were incubated for 28 h. The percentages of male and female gametocytes were calculated as follows: ([number of male or female gametocytes] ÷ [total number of schizonts plus male and female gametocytes]) × 100. Results are expressed as the mean ± SD of three independent experiments

The conserved *Plasmodium* protein PBANKA_0519900 was detected through IP-MS of 1019700::mCherry. Although RMgmDB suggests that parasite growth is delayed by PBANKA_0519900 deletion, the roles and cellular localization of PBANKA_0519900 remain unclear. Therefore, deletion mutants (Δ*0519900*) were generated by introducing gene-targeting vectors into the *P. berghei* ANKA genome (Additional file 1: Fig. S1D) and the roles of PBANKA_0519900 in the asexual development of malaria parasites were examined. Mice infected with Δ*0519900* mutants showed lower levels of parasitaemia than mice infected with control parasites (Fig. 4A) and their survival was prolonged compared to mice infected with control parasites (Fig. 4B). On the other hand, the percentages of both male and female gametocytes produced by Δ*0519900* parasites were comparable to those of control parasites (Fig. 4C, D).

### Cellular localization of PBANKA_0519900 in malaria parasites

Next, to investigate the cellular localization of PBANKA_0519900, transgenic parasites expressing the fusion proteins 0519900::GFP (green fluorescent protein) and 1019700::mCherry were generated (Additional file 1: Fig. S3A). In addition, transgenic parasites expressing the fusion proteins 1359300::GFP and 1019700::mCherry were generated as cytoplasmic controls, as VPS13 domain-containing proteins localize to the cytoplasm (Additional file 1: Fig. S3B). These GFP tags were introduced at the C-terminus of endogenous *PBANKA_0519900* or *PBANKA_1359300*. Expression of *0519900::gfp* and *1359300::gfp* was controlled by the corresponding endogenous native promoters. Both transgenic parasites were successfully generated (Additional file 1: Fig. S3).

**Fig. 5:**
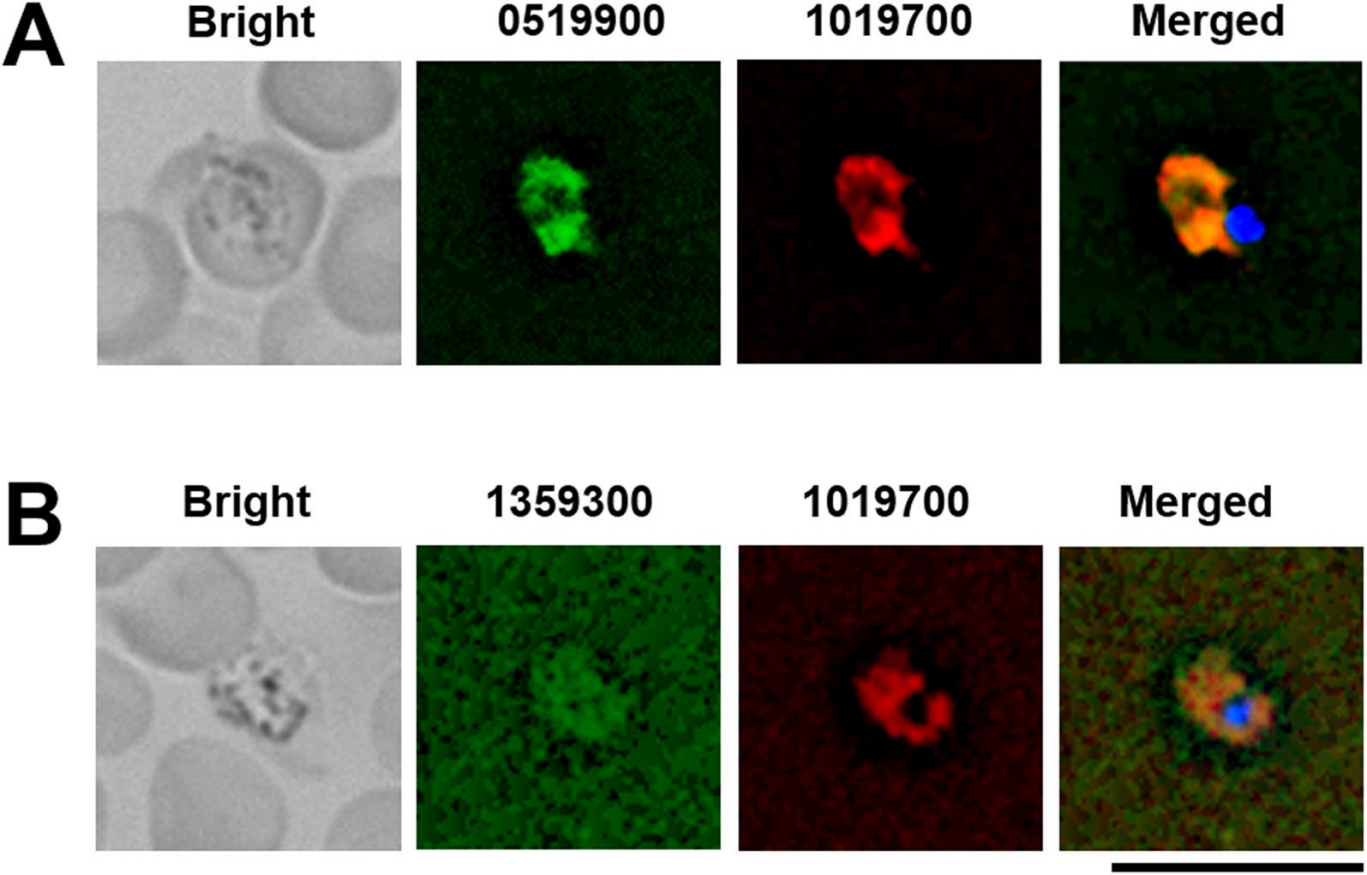
Cellular localization of PBANKA_0519900 and PBANKA_1359300 in *P. berghei* ANKA. Female B6 mice were infected with 1 × 10^4^ erythrocytes parasitized with transgenic *Plasmodium* expressing 0519900::GFP and 1019700::mCherry, or transgenic *Plasmodium* expressing 1359300::GFP and 1019700::mCherry. The cells’ nuclei were stained with Hoechst 33342 (blue). At least 50 parasitized erythrocytes were analysed, and the same fluorescence pattern was observed in trophozoites. **A** Transgenic parasites expressing 0519900::GFP (0519900, green) and 1019700::mCherry (1019700, red) during the trophozoite stage are shown. **B** Transgenic parasites expressing 1019700::mCherry (1019700, red) and 1359300::GFP (1359300, green) during the trophozoite stage are shown. Representative data are shown. Scale bar = 10 μm

To examine the cellular localization of 0519900::GFP and 1359300::GFP, live-cell fluorescence imaging of trophozoites was performed. As shown in Fig. 5, GFP signals were present in the cytoplasm in 0519900::GFP/1019700::mCherry-expressing mutants. GFP signals were low levels in 1359300::GFP/1019700::mCherry-expressing mutants, but the signals were mainly present in the cytoplasm. These findings suggest that PBANKA_0519900 and PBANKA_1359300 are cytoplasmic proteins. Taken together, these results demonstrate that the NTF2 domain-containing protein PBANKA_1019700 is a noncanonical NTF2-like superfamily protein.

## Discussion

The roles of PBANKA_1019700 during the asexual and sexual development stages of *P. berghei* ANKA were investigated using reverse genetics. The results showed that PBANKA_1019700 is not essential to either stage and is localized to the cytoplasm. Moreover, the IP-MS results for 1019700::mCherry indicated that PBANKA_1019700 does not interact with nuclear proteins. These results suggest that PBANKA_1019700 is not involved in mRNA export in malaria parasites.

In yeast, the Mex67/Mtr2 mRNA export receptor is required for mRNA export [1, 2]. Yeast Mex67 possesses an NTF2-like domain and interacts with adaptor proteins such as NAB2 [1, 3]. In *Plasmodium*, orthologs of adaptor proteins, such as NAB2, but not Mex67/Mtr2 have been identified. That PBANKA_1019700, which contains an NTF2-like domain, is the candidate protein of mRNA export receptor was supported by the results of the in silico analysis.

According to PlasmoDB, PBANKA_1019700 is expressed during the erythrocytic stage of the malaria parasite life cycle. However, the Rodent Malaria genetically modified Database (RMgmDB) contains no report on PBANKA_1019700 deletion. Meanwhile, the NTF2-like domain and the C-terminus of PBANKA_1019700 are conserved among *Plasmodium* species, including *Plasmodium falciparum.* Data from transposon screening of *P. falciparum* indicate that PF3D7_1423700, which possesses an NTF2-like domain, is not essential to the asexual development of *P. falciparum* (PlasmoDB).

Similar to PF3D7_1423700, PBANKA_1019700 is not essential to the asexual development of *P. berghei.* Moreover, in this study, adaptor proteins such as NAB2 were not detected through IP-MS of 1019700::mCherry whereas live-cell imaging showed that PBANKA_1019700 is a cytoplasmic protein. In addition, previous research suggested that NAB2 derived from *P. berghei* (PbNAB2) does not interact with any NTF2-like superfamily proteins [7].

Trypanosomes belong to the Discoba, a clade outside of the Opisthokonta [6]. In trypanosomes, an ortholog of yeast Mex67 has been characterized that is involved in mRNA export [23]. However, no orthologs of adaptor proteins, such as yeast NAB2, have been identified through bioinformatics in trypanosomes [24]. Although TbMex67 lacks the NTF2-like domain, it contains a CCCH-type zinc finger motif at its N-terminus [12]. The CCCH-type zinc finger motif of TbMex67 is essential for its interaction with the nuclear pore [12]. These findings indicate that an NTF2-like superfamily protein is not essential for mRNA export in parasitic unicellular eukaryotes.

In contrast to trypanosomes, orthologs of yeast Mex67 have not been identified in *Plasmodium*. However, *Plasmodium* possesses PbNAB2, which contains a CCCH-type zinc finger motif [7, 8]. In a previous study, IP-MS of PbNAB2::mCherry revealed that PbNAB2 interacts with PBANKA_1454600 [7], which was recently identified as the nucleoporin NUP269 [25], suggesting that PbNAB2 interacts directly with the nuclear pore to export mRNA. Together, these results indicate that proteins containing the CCCH-type zinc finger motif might directly interact with the nuclear pore and play a pivotal role in mRNA export in parasitic unicellular eukaryotes.

NTF2-like superfamily proteins possess both enzymatic and non-enzymatic functions, including mRNA export. The noncanonical NTF2-like protein ActVI-ORFA has been suggested to play an enzymatic role in polyketide biosynthesis in *Streptomyces* [26]. In this study, gametocyte production was reduced by PBANKA_1019700 deletion in *P. berghei*. IP-MS analysis of 1019700::mCherry showed that PBANKA_1019700 interacts with ubiquitin-related proteins and cytoplasmic PBANKA_0519900. In gametocytes, in which many biosynthetic pathways remain uncharacterized, the function of PBANKA_1019700 remains unclear. Our results thus far suggest that PBANKA_1019700 is a noncanonical NTF2-like superfamily protein associated with novel biosynthetic pathways, such as polyketide biosynthesis.

## Conclusion

PBANKA_1019700 is a noncanonical NTF2-like superfamily protein and that export of mRNA is independent of NTF2-like superfamily proteins in *Plasmodium*.

### Supplementary Information

**Additional file 1: Figure S1.** Schematic representation of the gene-targeting vector used to disrupt PBANKA_1019700, PBANKA_1101300 (SBP1) and PBANKA_0519900. **Figure S2.** Generation of parasites to investigate the localization of PBANKA_1019700.**Figure S3.** Generation of parasites to investigate the localization of PBANKA_0519900::GFP and PBANKA_1359300::GFP.

**Additional file 2: Table S1.** Sequence of primers used in this study.

**Additional file 3: Table S2.** Results of immunoprecipitation coupled to mass spectrometry in 1019700::mCherry parasites.

## Data Availability

All data generated or analysed during this study are included in this published article and its Additional files.

## Abbreviations

NTF2: Nuclear transport factor 2
IP-MS: Immunoprecipitation coupled to mass spectrometry
NAB2: Nuclear poly(A) binding protein 2
YRA1: Yeast RNA annealing protein
SR: Three serine/arginine-rich
NPL3: Nucleolar protein 3
GBP2: G-strand binding protein 2
HRB1: Hypothetical RNA-binding protein
SBP1: Skeleton binding protein 1
MAHRP1a: Membrane associated histidine-rich protein 1a
PSMs: Peptide spectral matches
