# Supplementary material for: Characterization of a nuclear transport factor 2-like domain-containing protein in Plasmodium berghei
Source: Malar J. 2024 Jan 9;23:13. doi: 10.1186/s12936-024-04839-9 (PMC10777651; doi:10.1186/s12936-024-04839-9)
Supplement: Supplementary file 1 — Additional file 1: Figure S1. Schematic representation of the gene-targeting vector used to disrupt PBANKA_1019700, PBANKA_1101300 (SBP1) and PBANKA_0519900. Figure S2. Generation of parasites to investigate the localization of PBANKA_1019700.Figure S3. Generation of parasites to investigate the localization of PBANKA_0519900::GFP and PBANKA_1359300::GFP. [file 12936_2024_4839_MOESM1_ESM.docx]

**Characterization of a nuclear transport factor 2-like domain-containing protein in *Plasmodium berghei***

Mamoru Niikura^1*^, Toshiyuki Fukutomi^2^, Jiro Mitobe^1^, Fumie Kobayashi^3^

^1^ Department of Infectious Diseases, Kyorin University School of Medicine, Tokyo, Japan

^2^ Department of Pharmacology and Toxicology, Kyorin University School of Medicine, Tokyo, Japan

^3^ Department of Environmental Science, School of Life and Environmental Science, Azabu University, Kanagawa 252-5201, Japan

**Additional figure and figure legends**

**
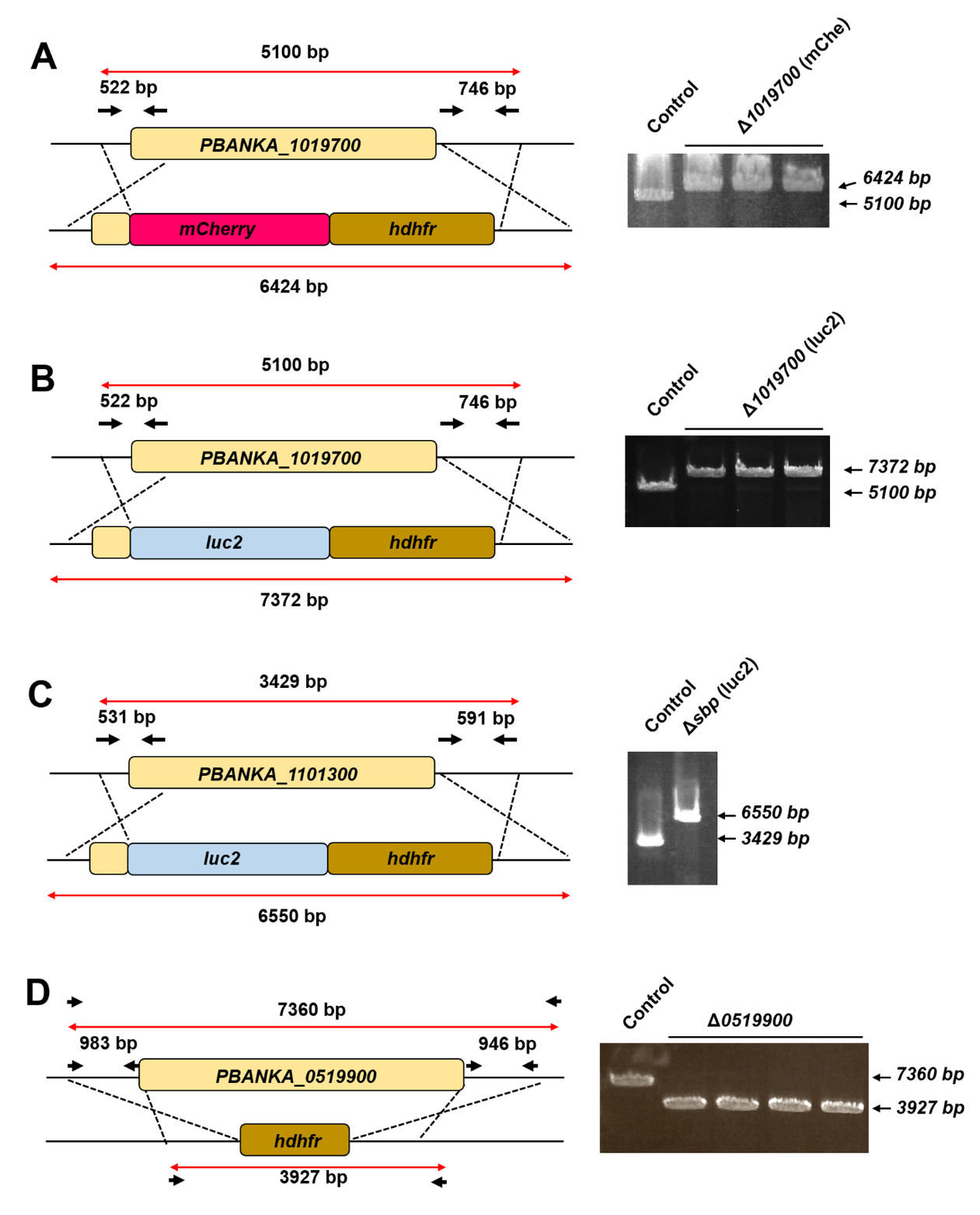
**

**Fig. S1 Schematic representation of the gene-targeting vector used to disrupt PBANKA_1019700,** **PBANKA_1101300 (SBP1) and PBANKA_0519900.**

Gene disruption was performed through double-crossover homologous recombination. The gene disruption vectors contained human dihydrofolate reductase–thymidylate synthase (*hdhfr*) and the 5′ and 3′ flanking regions of the target genes. *hDHFR* expression was controlled using the *elongation factor-1* (PBANKA_113340) promoter. *mCherry* and *luc2* expression were controlled by the *hsp70* (PBANKA_0711900) promoter. Arrows denote primers specific to the 5′ and 3′ regions of the target genes. (A) Introduction of the *mCherry-hDHFR*-expressing cassette into the *PBANKA_1019700* locus of wild-type (WT) *P. berghei* ANKA. Successful integration of the *mCherry-hDHFR*-expressing cassette into the *PBANKA_1019700* locus was confirmed through polymerase chain reaction (PCR; WT fragment, 5100 bp; Δ*1019700* fragment, 6124 bp). (B) Introduction of the *luc2-hDHFR*-expressing cassette into the *PBANKA_1019700* locus of WT *P. berghei* ANKA. Successful integration of the *luc2-hDHFR*-expressing cassette into the *PBANKA_1019700* locus was confirmed through PCR (WT fragment, 5100 bp; Δ*1019700* fragment, 7372 bp). (C) Introduction of the *luc2-hDHFR*-expressing cassette into the *PBANKA_1101300* locus of WT *P. berghei* ANKA. Successful integration of the *luc2-hDHFR*-expressing cassette into the *PBANKA_1101300* locus was confirmed through PCR (WT fragment, 3429 bp; Δ*1101300* fragment, 7372 bp). (D) Introduction of the *hDHFR*-expressing cassette into the *PBANKA_0519900* locus of WT *P. berghei* ANKA. Successful integration of the *hDHFR*-expressing cassette into the *PBANKA_0519900* locus was confirmed through PCR (WT fragment, 3927 bp; Δ*0519900* fragment, 7360 bp).


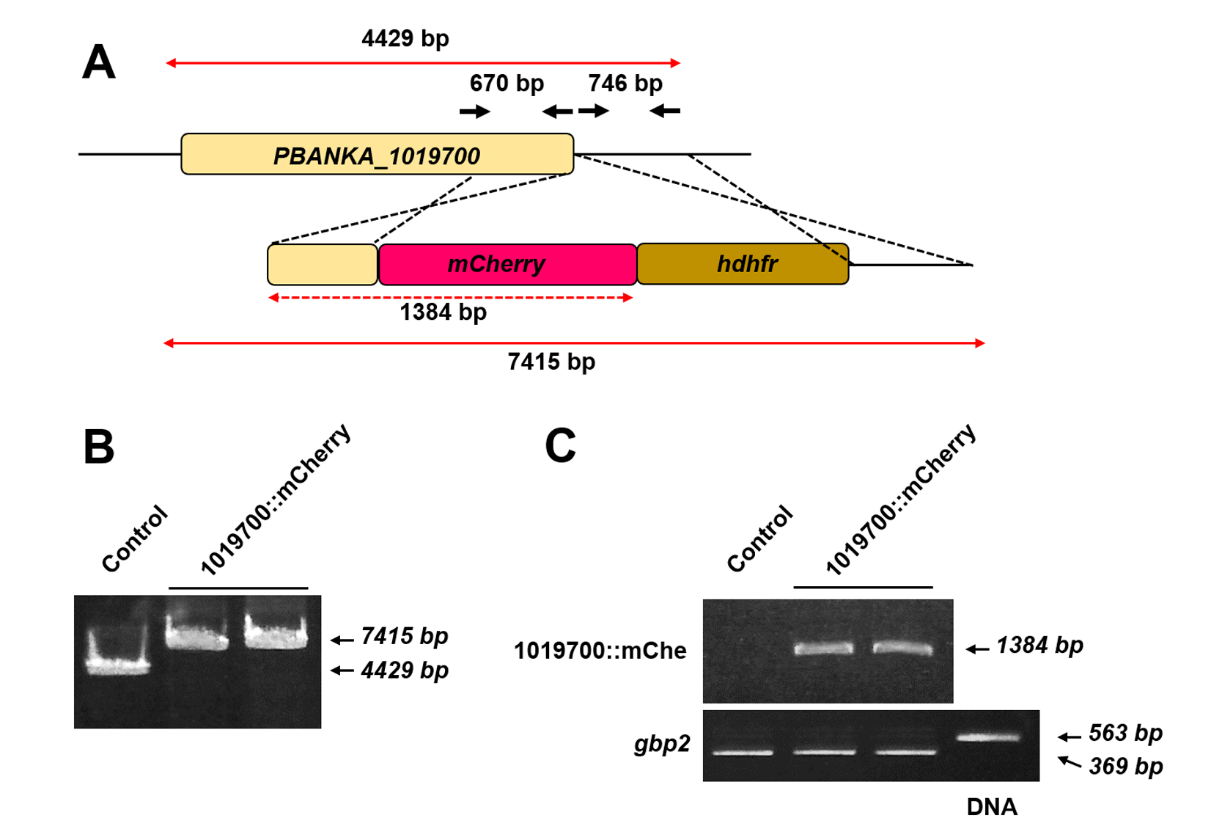


**Fig. S2 Generation of parasites to investigate the localization of PBANKA_1019700**

Schematic representation of the gene-targeting vectors used for *1019700::mCherry* expression. The gene-targeting vectors contained *mCherry*, *hdhfr*, and the 3′ regions of the target genes. The *1019700::mCherry* genes were under the control of their native promoters. *hdhfr* was under the control of the elongation factor-1 (PBANKA_113340) promoter. Arrows denote primers specific to the 5′ and 3′ regions of the gene-targeting vectors. (A) Introduction of the *1019700::mCherry* cassette into the 3′ region of the *gbp2* locus of wild-type (WT) *P. berghei* ANKA. (B) Successful integration of *mCherry* and *hdhfr* into the 3′ region of *PBANKA_1019700* was confirmed through PCR (WT fragment, 4429 bp; 1019700::mCherry fragment, 7415 bp). (C) Expression of *1019700::mCherry* and *gbp2* (PBANKA_1205000). Total RNA was isolated from 5 × 10^6^ packed red blood cells containing control parasites and 1019700::mCherry mutants. Expression of *1019700::mCherry* and *gbp2* was assessed via semi-quantitative reverse transcription PCR using specific primers (Table S1). *gbp2* was used as the positive control. DNA represents the control for DNA contamination of the RNA preparations.

**
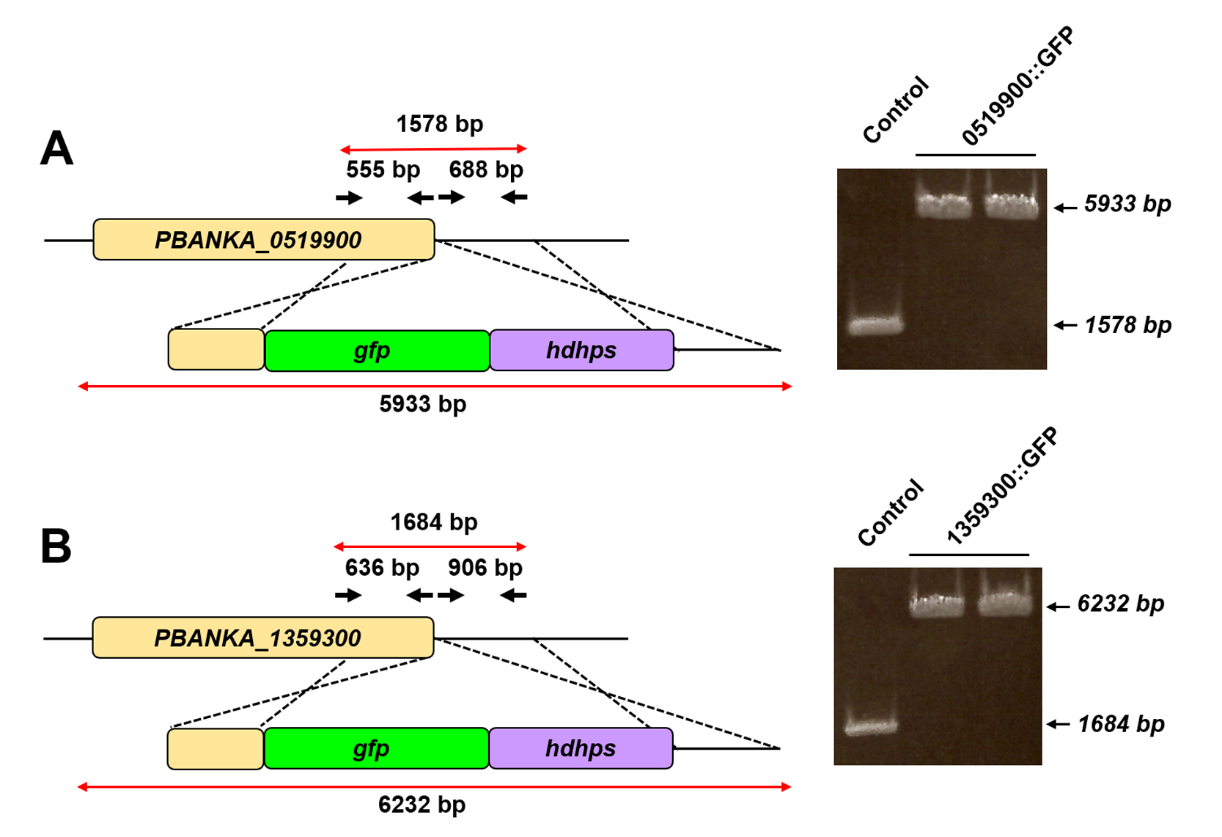
**

**Fig. S3 Generation of parasites to investigate the localization of PBANKA_0519900::GFP and PBANKA_1359300::GFP**

Schematic representation of the gene-targeting vectors used to express mCherry fused to 0519900::GFP and 1359300::GFP. The gene-targeting vector contained green fluorescent protein (*gfp*), mutated *human deoxyhypusine synthase* (*hdhps*), and the 3′ regions of the target genes. The *0519900::gfp* and *1359300::gfp* genes were under the control of their native promoters. *hdhps* was under the control of the elongation factor-1 (PBANKA_113340) promoter. Arrows denote primers specific to the 5′ and 3′ regions of the gene-targeting vectors. (A) Introduction of the *0519900::gfp* cassette into the 3′ region of the *PBANKA_0519900* locus of transgenic parasites expressing the fusion protein 1019700::mCherry. Successful integration of *gfp* and *hdhps* into the 3′ region of *PBANKA_0519900* was confirmed through PCR (control fragment, 1578 bp; 0519900::GFP fragment, 5933 bp). (B) Introduction of the *1359300::gfp* cassette into the 3′ region of the *PBANKA_1359300* locus of transgenic parasites expressing the fusion protein 1019700::mCherry. Successful integration of *gfp* and *hdhps* into the 3′ region of *PBANKA_1359300* was confirmed through PCR (control fragment, 1684 bp; 1359300::GFP fragment, 6232 bp).

**Table S1 Sequence of primers used in this study.**

**Table S2 Results of immunoprecipitation coupled to mass spectrometry in 1019700::mCherry parasites.**
